# Evaluation of addiction interventions: follow up after discharge

**DOI:** 10.1192/j.eurpsy.2025.981

**Published:** 2025-08-26

**Authors:** M. Perez Lopez, J. Chicharro

**Affiliations:** 1 SGA. Adicciones, Madrid-Salud. Ayuntamiento de Madrid, Madrid, Spain

## Abstract

**Introduction:**

Addiction treatments are complex, and their goals have changed over time. In the past, abstinence was the main objective. Nowadays, treatments focus on people and their recovery. They are developed to act in many areas of life, therefore appropriate measures are needed that really show the results achieved. One of the variables that has been shown to be appropriate for measuring the results of interventions is quality of life.

**Objectives:**

Our main objective was to evaluate the results of the intervention carried out on people who have been discharged, with an instrument that includes data on quality of life. Another objective was to standardize the measurement of results, establishing reliable criteria that include the diversity of people in treatment for addictions.

**Methods:**

A computer-assisted telephone survey was conducted, with 575 people, between May 2023 and June 2024. They had been for 3 or 6 months on therapeutic or voluntary discharge.

Criteria for therapeutic discharge, voluntary discharge, and abandonment were established. They included results in different areas: substance use, health and self-care, mental health, social/family integration and educational/work.

The World Health Organization Quality of Life BREF (WHOQOL BREF) questionnaire was used. Psychosocial and drug use questions were added to the evaluation.

**Results:**

At discharge, around 75% of people considered that they were in better health than before starting treatment, 60% believed that their quality of life was good or very good, 72% considered that their life had a lot or some meaning and 75% maintained abstinence from the substance for which they were in treatment.

Regarding quality of life (WHOQOL BREF) in people with discharge from treatment, the domain with the lowest score was the one of personal relationships.

Opiate patients were the ones with the lowest scores, with physical health values being highlighted. In terms of gender, women scored worse than men, especially in psychological health.

**Conclusions:**

The results obtained indicate that the biopsychosocial and interdisciplinary treatment of addictions at the Addictions Institute of Madrid City Council improves the overall quality of life and the perception of health. These changes are largely maintained over time, including abstinence.

**Disclosure of Interest:**

None Declared

